# Facets of Arctic Change

**DOI:** 10.1007/s13280-017-0952-4

**Published:** 2017-10-26

**Authors:** Jean-Claude Gascard, Anne-Sophie Crépin, Michael Karcher, Oran R. Young

**Affiliations:** 1 LOCEAN, University Pierre et Marie Curie, 4 Place Jussieu, 75005 Paris, France; 2 The Beijer Institute of Ecological Economics, The Royal Swedish Academy of Sciences, Lilla Frescativägen 4, Box 50005, 104 05 Stockholm, Sweden; 3 The Stockholm Resilience Centre, Stockholm University, Kräftriket 2 B, 10691 Stockholm, Sweden; 4 Ocean Atmosphere Systems GmbH, Tewessteg 4, 20249 Hamburg, Germany; 5 Alfred-Wegener-Institut Helmholtz-Zentrum für Polar- und Meeresforschung, Bussestrasse 24, 27570 Bremerhaven, Germany; 6 Bren School, University of California, Office Bren hall 4032, Santa Barbara, USA

This special issue highlights results from the project *Arctic Climate Change Economy and Society* (ACCESS, 2011–2015) supported within the Ocean of Tomorrow call of the European Union’s 7th framework programme. Focusing on the marine Arctic, ACCESS investigated climate impacts on marine transportation, seafood production, and the extraction of hydrocarbons up to 2050. The project dedicated particular attention to environmental sensitivities, sustainability, governance, strategic policy options, and management support.

A tight collaboration between experts with multiple disciplinary skills was necessary to derive and synthesize findings, to obtain an overall picture of the interactions between the different elements of the biophysical and socioeconomic systems and to evaluate climate change impacts in the Arctic Ocean. The ACCESS project convened around 100 researchers from 27 different partner institutions in ten different European countries. Researchers’ disciplinary backgrounds covered a wide range of natural and social sciences, including economics, social anthropology, systems ecology, marine biology, climatology and law; they came from universities, national research centres and small and medium enterprises. Stakeholders from local and indigenous populations, industry and non-governmental organizations were also involved. Necessarily, the project had to employ manifold approaches and methods. Participants gathered environmental observations from satellites, buoys, temporary observatories stationed on drifting ice platforms, and measurements of chemical and noise pollution. Societal and economic information was collected from databanks, through semi-structured interviews, and behavioural experiments. ACCESS researchers used a variety of models to simulate and better understand the past, and evaluate expected changes in natural and socioeconomic systems under future conditions; they also performed controlled sensitivity experiments of the natural and human systems. New methods and tools were developed such as a marine spatial planning tool, an integrated ecosystem-based management framework, and numerical modelling approaches to improve ship routing in ice-covered waters or to optimize observational networks. In addition, the project hosted meetings with stakeholders and carried out various dissemination activities, including two summer schools to train young scientists in cross-sectoral approaches, workshops, and a series of newsletters and policy briefs. A website is maintained beyond the lifetime of the project, to serve as a hub for project-related information and the projects’ products (www.access-eu.org).

We started synthesizing results from ACCESS 2 years after the start of the project through cross-sectoral sessions organized during the annual general assembly meetings and meetings including researchers from different disciplines organized back-to-back with the summer schools held in 2013 and 2014. Communication and trust were essential ingredients for the success of such a large and complex research project. Barriers to communication included physical distance and different spoken and scientific languages, not only among participants from different countries but also among different disciplines and scientific cultures. Ensuring a true collaborative spirit required patience and open-mindedness among all participants. In addition to the participating scientists, an advisory board, including Adele Airoldi, Inuuteq Holm Olsen, Hannu Hallinen, Hajo Eicken, and guest editor Oran Young, provided valuable advice at all stages of the project dealing with science, politics, and indigenous peoples’ perspective.

ACCESS produced more than 100 scientific deliverables between March 2011 and February 2015. Most of the ACCESS results are included in this special issue or in other scientific peer-reviewed publications which are cited in this issue.

We hope you will enjoy the reading!

